# Investigating the role of defense mechanisms on marital adjustment in infertile couples based on the cause of infertility: A cross-sectional study

**DOI:** 10.18502/ijrm.v21i2.12802

**Published:** 2023-03-08

**Authors:** Behnaz Navid, Farideh Malekzadeh, Maryam Mohammadi, Reza Omani-Samani

**Affiliations:** ^1^Department of Psychology, Payame Noor University, Tehran, Iran.; ^2^Department of Endocrinology and Female Infertility, Reproductive Biomedicine Research Center, Royan Institute for Reproductive Biomedicine, ACECR, Tehran, Iran.; ^3^Reproductive Epidemiology Research Center, Royan Institute for Reproductive Biomedicine, ACECR, Tehran, Iran.; ^4^Department of Medical Ethics and Law, Reproductive Biomedicine Research Center, Royan Institute for Reproductive Biomedicine, ACECR, Tehran, Iran.

**Keywords:** Defense mechanism, Marital relationship, Infertility.

## Abstract

**Background:**

Infertility affects individual's and couples' adjustment. The defense mechanism and marital adjustment are mental processes that play a key role in infertile couples' life.

**Objective:**

This study aims to investigate the role of defense mechanisms on marital adjustment in infertile couples based on the cause of infertility.

**Materials and Methods:**

This cross-sectional study was conducted on 400 infertile couples at Royan Institute (A referral center for infertility, Tehran, Iran). Infertile couples were divided into 4 groups based on the cause of infertility (female, male, both, and unknown). Demographic questionnaire, revised dyadic adjustment scale, and defense styles questionnaire were used for data collection.

**Results:**

Results showed that the mean score of marital adjustment of men based on the cause of infertility (female, male, and both) was significantly higher in comparison with their wives (p = 0.04, p ≤ 0.001, p ≤ 0.001, respectively). However, no difference was observed between the mean score of women, men, and couples (women and husbands) in defense mechanisms. But marital adjustment has a significant positive correlation with mature defense mechanisms and a negative correlation with immature ones (p < 0.05).

**Conclusion:**

The mean score of marital adjustment is higher in men than in their wives in all groups. So, counseling infertile couples in the field of marital adjustment and training in the use of mature defense mechanisms is recommended especially in women.

## 1. Introduction

Infertility can cause guilt and shame sense in couples which results in changes in the plan of their family identity, marital stability, and marital status (1, 2). Childlessness is a critical and stressful situation for couples of reproductive ages (2). Infertility can affect the physical, psychological, social, and economic wellness of couples, which leads to tension in personal and interpersonal relationships (3). The results of a meta-analysis study revealed that the scores of depression and anxiety in infertile couples were higher than the fertile ones, and this difference has been observed in many national and international studies (4). It has also been shown that the level of depression in infertile women and men was higher than the healthy ones (5).

Numerous studies have shown that infertile couples have lower marital satisfaction, expression of interest in their partner, marital adjustment, and sexual satisfaction than the fertile ones (6, 7). Marital adjustment consists of marital satisfaction, cohesion, agreement, and affection. Also, it affects the physical and mental health of family members (8, 9). Marital adjustment is “the integration of the couple in a union, in which the 2 personalities are not merely merged or sub-merged but interact to complement each other for mutual satisfaction and the achievement of common objectives” (9). Adjustments in marital relationships can cause significant improvements in quality of life among infertile couples (10).

Infertile couples that are undergoing infertility treatment apply many psychological mechanisms for coping with the stress due to this process; therefore, recognizing these mechanisms may be helpful in diagnosing their problems, understanding the type of treatment, coping and overcoming the barriers, and facilitating the treatment (11, 12).

Defense mechanisms occur unconsciously that can be used as an indicator for the level of individual adaptation with respect to significant psychological challenges (13). It should be noted that defense mechanisms may lead to compromising problems. This is due to neglecting the facts causing spousal relationship issues. However, focusing on facts and mutual needs between partners can be achieved by using effective and adaptive defense mechanisms (2). Defense mechanisms are divided by valliant into levels of mature, immature, and neurotic defenses (14). A study reported that infertile couples use immature defense mechanisms more than fertile ones (15). Another study indicated that women with sexual dysfunction are more likely to use immature and neurotic defense mechanisms than normal women and individuals' sexual health is significantly related to their defense mechanisms. Therefore, defense mechanisms have the capacity to be considered as one of the therapeutic variables and interventions (16).

In this study, we hypothesized that the cause of infertility can influence the type of defense mechanisms used by infertile couples and the marital adjustments made by them. To the best of our knowledge, this was the first study on the marital adjustment and defense mechanisms in both husbands and wives (couples) with infertility, based on its cause.

## 2. Materials and Methods

This cross-sectional study was conducted at Royan Institute, a referral center for infertility treatment in Tehran, Iran from April-December 2019. Considering that the main study of the project was the comparison of the mean scores of the studied variables between 4 groups, the analysis of variance test was used. To determine the sample size, type I error 0.05 and type II error 0.2 (power 0.8) were considered. Also, according to the previous research and the researcher's expectation of the practical (or clinical) difference, the effect size was equal to 0.126, and the sample size was 694. Including missing data, 700 people were considered. We used sequential sampling which is one of the non-random sampling methods. They were divided into 4 groups based on the cause of infertility (female, male, both, and unknown). First, the researcher explained the aim of the study and then the couples were requested to fill out 3 questionnaires separately (man and woman) at Royan Institute. The inclusion criteria were: a) Couples (men and women) 18-45 yr old; b) Diagnosed as infertile couples by Royan Institute; c) Those having the ability to read, write, and comprehend Persian. The exclusion criteria were: incomplete questionnaires and dissatisfaction with participating in the study, infertile couples who had severe psychiatric disorders (such as severe depression or psychosis), and were on medication were excluded from the study. In this study, 3 questionnaires had been used to collect data.

### Demographic and fertility characteristics

The questionnaire included age, sex, education, infertility duration, cause of infertility, and history of abortion.

### Revised dyadic adjustment scale (RDAS)

The RDAS is a 14-item scale that assesses marital adjustment. The RDAS questionnaire consists of 3 subscales, including consensus, satisfaction, and cohesion endorsed on a 6-point Likert scale, in which participants are asked to show their response on a 6-point scale ranging from 0 = disagreement to 5 = always agreement. In sum, a higher score indicates greater relationship adjustment (17). The Cronbach's alpha coefficients in previous studies have been reported from 0.80-0.90 (18). In Iran, Cronbach's alphas for this questionnaire was 0.92, demonstrating the RDAS that could be used to measure a clinically significant change in individuals (19).

### Defense styles questionnaire (DSQ)

The DSQ was simplified into 40 questions related to 20 defense mechanisms (20) divided by valliant into levels of immature, neurotic, and mature (14). The internal consistency of DSQ in the mature, neurotic, and immature sections were 0.70, 0.61, and 0.83 respectively (21). This questionnaire was normalized in Iran with Cronbach's alpha 0.716. The scoring scale of DSQ is Likert from 1-5 which is shown by 1 strongly disagree and 5 strongly agree (22).

### Ethical considerations

The aim of the study and the confidentiality of the data were clearly explained for all participants. Therefore, completing the questionnaire by couples is considered as informed consent. The Ethics Committee of Royan Institute, Tehran, Iran approved this study (Code: IR.ACECR.ROYAN.REC.1396.148).

### Statistical analysis

Statistical analysis was performed using the Statistical Package for the Social Sciences. SPSS version 22 (Inc. Chicago, IL, USA). Continuous variables were expressed as mean ± standard deviation (SD) and categorical variables as frequencies (percentage). Normality of the variables was checked with Kolmogorov-Smirnov Test. Pearson correlation coefficient was used to examine the relationship between study variables and paired *t* test was used to evaluate the difference between male and female data (wives and husbands) and used variance analysis to evaluate the difference between groups of infertility (female factor, male factor, both factor, and unknown factors). Finally, multiple linear regression analysis was performed by controlling confounders. P-value < 0.05 was considered statistically significant.

## 3. Results

In this study, 400 infertile couples consisting of 400 (50%) men and 400 (50%) women participated with a mean age of 33.37 ± 5.17 yr for males and 30.37 ± 5.51 for females. Among the participants, 430 individuals (53.75%) had under diploma/diploma, 368 (46%) academic education, and 2 missing educational information (0.25%). The cause of infertility in couples was 155 (38.7%) male, 75 (18.7%) female, 62 (15.5%) both, and 108 (27.1%) unknown factors. The mean duration of infertility was 4.68 ± 3.54 yr. Also, 88 (22.1%) of the participants reported a history of abortion.

### Univariate analysis

Table I shows the relationship between the subscales of defense mechanism with the cause of infertility in women and their husbands.

### Immature defenses mechanism subscales

The mean score for the projection was significantly lower in men than their wives in the female and unknown factors (p ≤ 0.001, p = 0.02, respectively). The mean score of denial in all causes of infertility in men was significantly higher than their wives (p < 0.05). Devaluation in couples with the male factor, the mean score of men was significantly higher than their wives (p = 0.03), but in both factors, women had a higher score than their husbands (p ≤ 0.001). Also, women and men with female factors had a significantly higher score of devaluation than other groups of causes of infertility (p ≤ 0.001, p = 0.02, respectively). The mean score of acting out in women with female and male factors was higher than their husbands (p ≤ 0.001, p = 0.01, respectively). In the somatization section in all groups of causes of infertility, women had higher scores than their husbands (p < 0.05). In passive aggression, women had higher scores than their husbands in unknown causes of infertility (p = 0.03). The score of displacement in infertile women with both factors was higher than in other groups (p = 0.01). The score of splitting among women was lower than their husbands with male and unknown factors (p ≤ 0.001, p = 0.04). The total score of immature defenses mechanism was not different between women and men in all groups of infertility factors (p > 0.05).

### Mature defense mechanism subscales

The mean score of sublimation was higher in women than their husbands in the unknown factors (p = 0.02). In female and unknown factors, humor was higher in men than in their wives (p = 0.02, p = 0.04, respectively). But, humor in women with male factor was higher than other groups (p = 0.02). The mean score of anticipation in female and male factors was lower in women than their husbands (p = 0.05, p ≤ 0.001, respectively). The total score of mature defenses mechanism was not different between women and men in all groups (p > 0.05). But, in men with male factors, the mean score of mature defenses mechanism was higher than their wives nearly significant (p = 0.06).

### Neurotic defenses mechanism subscales

Pseudoaltruism score was more in women than their husbands (p ≤ 0.001). Men had more mean scores of idealization than their wives in male and unknown factors (p ≤ 0.001). The total score of neurotic defenses mechanism was not different between women and men in all groups of factors (p > 0.05).

As seen in table II, in the total and subscale of marital adjustment, the consensus was higher in men than their wives in both and female factors (p = 0.03, p = 0.05, respectively). Satisfaction was higher in men than their wives in female, male, and both factor groups (p < 0.05). However, women in the unknown factor group had higher satisfaction than other groups (p ≤ 0.001). The cohesion of men was higher than their wives in both factor group (p = 0.04). The total score of marital adjustment in all groups were lower in women than their husbands which were significant (p < 0.05) except in the unknown factor group (p = 0.29).

### Correlations among study variables 

Bivariate correlations were conducted among subscales of 2 questionnaires (marital adjustment and defense mechanism), as shown in table III. The total score of the marital adjustment questionnaire and its subscales (included consensus, satisfaction, cohesion) with the immature defense mechanism had a negative significant correlation. Also, the total score of marital adjustment and their subscales (including satisfaction and cohesion) had a positive significant correlation with mature defense mechanism (p < 0.05).

### Multiple linear regression analysis

For the marital adjustment, the used method was a covariate selection method, in step 1, demographics and subscales of defense mechanism entered to model, sex and immature defense, mature defense, and neurotic defense which were significantly related to marital adjustment (B = -1.94, p ≤ 0.001 and B = -2.30, p ≤ 0.001 and B = 1.51, p ≤ 0.001 and B = 0.57, p = 0.04, respectively). When sex, education, cause of infertility, duration of infertility, and subscales defense mechanism were in the marital adjustment model, the model adjusted R^2^ was equal to 0.13. On the other hand, variance inflation factor and tolerance of variables showed the model was not collinear.

In step 2, sex and immature defense, mature defense, and neurotic defense entered to model mature and neurotic defense were positively correlated with the marital adjustment (B = 1.45, p ≤ 0.001 and B = 0.55, p ≤ 0.001 respectively) and sex and immature defense were negatively correlated with the marital adjustment (B = -1.47, p ≤ 0.001 and B = -2.44, p ≤ 0.001 respectively). When sex, immature defense, mature defense, and neurotic defense were in the model, there was an improvement in the model (adjusted R ${}^{2}$ = 0.21, p ≤ 0.001, Table IV).

**Table 1:** Defense mechanism subscales in participants (n = 800)


**Defense mechanism**			**Female factor**	**Male factor**	**Both factor**	**Unknown factor**	**P-value ${}^{\text{a}}$ **
**Immature defenses**							
	**Rationalization**						
	**Female**	13.89 ± 2.65	14.20 ± 2.35	13.87 ± 2.67	14.30 ± 2.23	0.56
	**Male**	14.29 ± 2.85	14.06 ± 2.71	14.29 ± 2.81	14.23 ± 2.69	0.91
	**P-value ${}^{\text{b}}$ **	0.42	0.75	0.39	0.95	
**Immature defenses**							
	**Projection**						
	**Female**	9.90 ± 4.06	8.31 ± 4.04	9.17 ± 4.30	8.81 ± 4.37	0.05*
	**Male**	7.87 ± 3.80	7.45 ± 3.90	8.41 ± 3.61	7.47 ± 4.16	0.36
	**P-value ${}^{\text{b}}$ **	≤ 0.001*	0.06	0.20	0.02*	
	**Denial**						
	**Female**	7.90 ± 4.00	7.90 ± 3.94	7.45 ± 3.69	8.67 ± 3.96	0.21
	**Male**	9.87 ± 4.34	9.98 ± 4.04	9.54 ± 3.67	9.80 ± 3.87	0.90
	**P-value ${}^{\text{b}}$ **	≤ 0.001*	≤ 0.001*	0.01*	0.03*	
	**Omnipotence**						
	**Female**	11.57 ± 3.91	11.74 ± 3.25	11.16 ± 3.72	12.17 ± 3.50	0.32
	**Male**	12.27 ± 3.88	12.33 ± 3.14	12.27 ± 3.71	12.42 ± 3.33	0.99
	**P-value ${}^{\text{b}}$ **	0.23	0.11	0.11	0.53	
	**Devaluation**						
	**Female**	11.33 ± 3.64	9.64 ± 3.44	10.61 ± 3.26	9.89 ± 3.66	≤ 0.001*
	**Male**	10.67 ± 3.79	10.41 ± 3.62	8.91 ± 3.63	10.13 ± 3.52	0.02*
	**P-value ${}^{\text{b}}$ **	0.18	0.03*	≤ 0.001*	0.56	
	**Acting out**						
	**Female**	12.83 ± 3.84	11.87 ± 3.93	11.38 ± 3.87	11.87 ± 3.81	0.16
	**Male**	11.05 ± 4.03	10.58 ± 4.55	10.82 ± 4.17	11.15 ± 4.50	0.74
	**P-value ${}^{\text{b}}$ **	≤ 0.001*	0.01*	0.27	0.25	
	**Somatization**						
	**Female**	14.52 ± 3.67	13.25 ± 3.56	13.59 ± 3.14	13.51 ± 3.57	0.09
	**Male**	11.78 ± 4.34	11.85 ± 4.29	12.09 ± 4.19	10.87 ± 4.33	0.21
	**P-value ${}^{\text{b}}$ **	≤ 0.001*	≤ 0.001*	0.02*	≤ 0.001*	
	**Autistic fantasy**						
	**Female**	10.49 ± 5.23	10.30 ± 4.74	10.43 ± 4.70	10.12 ± 4.80	0.93
	**Male**	9.47 ± 4.89	9.38 ± 4.71	9.51 ± 4.40	10.14 ± 4.48	0.81
	**P-value ${}^{\text{b}}$ **	0.97	0.53	0.20	0.46	
	**Dissociation**						
	**Female**	10.68 ± 3.76	10.03 ± 3.67	10.43 ± 3.32	10.64 ± 4.19	0.50
	**Male**	10.87 ± 4.23	10.27 ± 3.89	10.75 ± 4.29	10.30 ± 4.01	0.66
	**P-value ${}^{\text{b}}$ **	0.75	0.42	0.80	0.52	
	**Passive aggression**						
	**Female**	9.87 ± 3.69	9.25 ± 3.82	10.32 ± 3.92	9.92 ± 4.15	0.26
	**Male**	10.45 ± 4.29	9.07 ± 3.96	9.37 ± 4.07	8.93 ± 3.80	0.06
	**P-value ${}^{\text{b}}$ **	0.36	0.75	0.11	0.03*	
**Immature defenses**							
	**Displacement**					
	**Female**	10.04 ± 4.29	8.48 ± 3.88	10.14 ± 4.39	8.82 ± 4.49	0.01*
	**Male**	8.43 ± 4.14	8.67 ± 4.33	9.67 ± 4.44	8.30 ± 3.65	0.19
	**P-value ${}^{\text{b}}$ **	0.01*	0.61	0.47	0.27	
	**Splitting**					
	**Female**	8.89 ± 4.61	7.74 ± 4.09	8.35 ± 4.31	8.09 ± 4.45	0.30
	**Male**	9.29 ± 4.17	9.50 ± 4.31	8.62 ± 4.16	9.25 ± 4.33	0.60
	**P-value ${}^{\text{b}}$ **	0.51	≤ 0.001*	0.85	0.03*	
**Mature defenses**							
	**Suppression**						
	**Female**	11.92 ± 3.71	11.64 ± 3.59	11.24 ± 4.05	11.67 ± 3.68	0.76
	**Male**	12.06 ± 3.85	12.20 ± 3.45	12.50 ± 3.25	11.43 ± 3.59	0.21
	**P-value ${}^{\text{b}}$ **	0.82	0.11	0.07	0.54	
	**Sublimation**						
	**Female**	11.96 ± 3.81	11.44 ± 3.47	11.88 ± 3.84	11.68 ± 3.81	0.74
	**Male**	11.52 ± 3.33	11.51 ± 3.59	11.19 ± 3.97	10.62 ± 3.64	0.22
	**P-value ${}^{\text{b}}$ **	0.47	0.74	0.22	0.02*	
	**Humor**						
	**Female**	11.25 ± 3.96	12.69 ± 3.06	12.27 ± 3.23	12.08 ± 3.32	0.02*
	**Male**	12.71 ± 3.68	12.90 ± 3.34	13.04 ± 3.61	12.91 ± 3.25	0.95
	**P-value ${}^{\text{b}}$ **	0.02*	0.51	0.15	0.04*	
	**Anticipation**						
	**Female**	14.41 ± 2.94	14.35 ± 2.53	14.36 ± 3.07	14.63 ± 2.60	0.60
	**Male**	15.25 ± 2.16	15.12 ± 2.20	14.93 ± 2.20	14.77 ± 2.60	0.50
	**P-value ${}^{\text{b}}$ **	0.04*	≤ 0.001*	0.08	0.72	
**Neurotic defenses**							
	**Pseudoaltruism**						
	**Female**	14.38 ± 3.05	13.72 ± 2.77	14.61 ± 2.38	14.00 ± 3.02	0.14
	**Male**	13.70 ± 3.12	13.57 ± 3.19	13.35 ± 3.28	13.97 ± 2.78	0.60
	**P-value ${}^{\text{b}}$ **	0.11	0.80	≤ 0.001*	0.98	
	**Reaction formation**						
	**Female**	10.60 ± 4.21	10.24 ± 3.74	10.16 ± 3.83	10.06 ± 4.15	0.84
	**Male**	10.41 ± 4.62	10.15 ± 4.26	10.09 ± 3.97	10.34 ± 4.08	0.95
	**P-value ${}^{\text{b}}$ **	0.79	0.93	0.79	0.64	
	**Idealization**						
	**Female**	12.61 ± 3.57	13.29 ± 3.33	12.29 ± 3.53	13.26 ± 3.12	0.13
	**Male**	12.05 ± 3.71	11.98 ± 4.10	12.33 ± 3.61	11.70 ± 4.00	0.83
	**P-value ${}^{\text{b}}$ **	0.31	≤ 0.001*	0.87	≤ 0.001*	
	**Undoing**					
	**Female**	12.04 ± 3.00	12.22 ± 3.42	12.30 ± 3.44	11.85 ± 3.40	0.87
	**Male**	12.55 ± 3.58	11.96 ± 3.88	12.38 ± 3.56	11.96 ± 3.77	0.64
	**P-value ${}^{\text{b}}$ **	0.32	0.55	1.00	0.79	
Data presented as Mean ± SD. P-value ${}^{\text{a}}$ , One way ANOVA, p-value ${}^{\text{b}}$ , Paired *t* test and *p < 0.05							

**Table 2:** Marital adjustment subscales participants (n = 800)


**Marital adjustment**		**Female factor**	**Male factor**	**Both factor**	**Unknown factor**	**P-value ${}^{\text{a}}$ **
**Consensus**						
	**Female**	24.84 ± 3.63	24.70 ± 4.37	24.09 ± 3.36	24.91 ± 3.80	0.60
	**Male**	25.79 ± 3.41	25.43 ± 3.00	25.08 ± 3.79	25.00 ± 3.73	0.41
	**P-value ${}^{\text{b}}$ **	0.05*	0.07	0.03*	0.75	
**Satisfaction**						
	**Female**	15.30 ± 2.61	15.36 ± 2.91	14.29 ± 3.22	15.86 ± 2.59	≤ 0.001*
	**Male**	16.03 ± 1.88	16.35 ± 2.42	15.87 ± 2.35	16.01 ± 2.84	0.50
	**P-value ${}^{\text{b}}$ **	0.02*	≤ 0.001*	≤ 0.001*	0.54	
**Cohesion**						
	**Female**	9.22 ± 4.30	10.11 ± 4.49	9.25 ± 4.34	9.55 ± 4.20	0.39
	**Male**	9.46 ± 4.20	10.12 ± 4.19	10.43 ± 4.25	10.07 ± 4.47	0.58
	**P-value ${}^{\text{b}}$ **	0.59	0.93	0.04*	0.32	
**Total marital adjustment**						
	**Female**	49.37 ± 7.94	50.18 ± 8.84	47.64 ± 8.34	50.33 ± 7.66	0.17
	**Male**	51.28 ± 7.46	51.96 ± 6.87	51.39 ± 8.20	51.09 ± 7.78	0.80
	**P-value ${}^{\text{b}}$ **	0.04*	≤ 0.001*	≤ 0.001*	0.30	
Data presented as Mean ± SD. P-value ${}^{\text{a}}$ , One way ANOVA, p-value ${}^{\text{b}}$ , Paired *t* test. * p < 0.05						

**Table 3:** The relationship of marital adjustment subscales with defense mechanism subscales


	**Consensus**	**Satisfaction**	**Cohesion**	**Marital adjustment**	**Immature defenses**	**Mature defenses**	**Neurotic defenses**
**Consensus**	- 0.42*	0.29*	0.76*	-0.10*	0.18	0.01
**Satisfaction**	- 0.27*	0.68*	-0.24*	0.07*	-0.04
**Cohesion**		- 0.78*	-0.20*	0.10*	0.01
**Marital adjustment**		- -0.24*	0.16*	0
**Immature defenses**			- 0.22*	0.46*
**Mature defenses**			- 0.38*
**Neurotic defenses**				-
Data are presented as a Pearson correlation coefficient for relationship marital adjustment subscales with defense mechanism subscales and Pearson correlation coefficient and, *p < 0.05							

**Table 4:** The results of hierarchical model selection in the multiple linear regressions, including factors related to the total score of marital adjustment


	**Marital adjustment**			
	**B**	**SE**	**Wald**	**P-value**
**Step 1:**				
**Sex (female vs. male)**	-1.94	0.56	11.87	≤ 0.001*
**Education (Educated vs. under diploma/diploma)**	-0.60	0.06	0.97	0.32
**Duration of infertility (Year)**	-0.02	0.08	0.09	0.76
**Age (Year)**	-0.08	0.05	2.61	0.11
**Cause of infertility (unknown vs. female/male/both)**	-1.11	0.83	1.79	0.18

## 4. Discussion

In the present study, we aimed to investigate the role of defense mechanisms on marital adjustment in infertile couples referred to Royan Institute based on the cause of infertility.

Our results showed that the mean score of marital adjustment (total and subscales) in all groups was higher in men than in their wives, which was supported by previous studies (23, 24). In women with male infertility factors, the rate of marital satisfaction was more than those with female infertility factors (25). A systematic review in 2012 reported that the marital relationship in infertile men with the diagnosis of infertility was not seriously impaired (24). The difference in marital adjustment in fertile and infertile women is controversial. Some studies reported that marital adjustment in fertile women was higher than infertile ones (15, 24). Although, others believe that there was no significant difference in the total score of marital satisfaction between fertile and infertile women (26), infertility does not reduce marital satisfaction in infertile women (27). The mean score of marital adjustment in infertile women with unknown factor group were higher in other groups (male, female, both). Perhaps it may be explained that in cultures and societies, women are considered to be responsible for infertility and this issue affects other aspects such as their life or marital satisfaction. But, when the cause of infertility is unknown, women might feel less guilty, and it could increase marital satisfaction in them.

Also, our results indicated marital adjustment was negatively correlated with sex (female vs. male) which was not related to some of the demographic features such as (age, infertility duration, and history of abortion). Another study reported a positive correlation between marital satisfaction with age and infertility duration (28). It means adjustment with each stressful situation such as chillness takes time to resolve their problems in this time. Another study reported that marital adjustment was negatively correlated with emotional disorders (29). In addition to results, our study revealed that defense mechanisms affect marital adjustment. It seems that the mature defense mechanisms had a significant positive association with marital adjustment. This means that infertile couples using the more mature defense mechanisms when exposed to stressful events, have more adjusted behavior and marital adjustment in their life (30). Also, there was a negative significant relationship between immature defense mechanisms and marital adjustment; this means by increasing the use of immature defense mechanisms, depression and stressful behavior happen more often happen, decreasing marital satisfaction.

Other research has revealed that there is a significant relationship between immature defense mechanisms and lower psychological and also physical health such as depression and stress. However, mature defense mechanisms are associated with mental and physical health and more life adjustment in adults (31). According to recent studies, using mature defense mechanisms predicts psychological adjustment and physical health (32).

Based on the present study findings, infertile men (in all groups) used denial mechanisms more than their wives. Women also used the somatization mechanism more than their husbands (in all groups). To justify this, it can be said that men by avoiding a stressful situation, try to resolve this subject without any change and use denial mechanism and they also tend to turn negative emotions into physical symptoms in stressful events so they use the somatization mechanism. These findings were consistent with the research results (33, 34).

According to studies, infertile females using defense mechanisms were more than fertile ones which affected the women's defense mechanism score, and also use of immature defense mechanisms were more in fertile women (15, 34, 35).

### Limitations

Our study has several limitations. First, we relied on patients coming to only one center, but it is a referral clinic for infertility treatment in Iran and patients come to this center from all around the country. Second, the cross-sectional nature of the study only allows no conclusions on causality and just reveals a correlation. Finally, this research depended on self-report findings, and information from other sources could not be obtained to confirm the findings of the self-report.

## 5. Conclusion

Understanding psychological defense mechanisms used by infertile persons may be helpful in the diagnosis of various problems. Marital adjustment is directly related to mature defense mechanisms. Also, the mean score of marital adjustment is higher in men than in their wives in all groups. It seems that infertile women are more vulnerable to its psychological consequences than infertile men. In sum, according to the results of this study, counseling infertile couples in the field of marital adjustment and training in the use of mature defense mechanisms, especially in women is recommended.

##  Conflict of Interest

The authors report no conflict of interest. 
